# Expression of concern: Upregulation of lncRNA SUMO1P3 promotes proliferation, invasion and drug resistance in gastric cancer through interacting with the CNBP protein

**DOI:** 10.1039/d2ra90005j

**Published:** 2022-02-05

**Authors:** Laura Fisher

**Affiliations:** Royal Society of Chemistry Thomas Graham House, Science Park, Milton Road Cambridge CB4 0WF UK advances-rsc@rsc.org

## Abstract

Expression of concern for ‘Upregulation of lncRNA SUMO1P3 promotes proliferation, invasion and drug resistance in gastric cancer through interacting with the CNBP protein’ by Yinmou Guo *et al.*, *RSC Adv.*, 2020, **10**, 6006–6016, DOI: 10.1039/C9RA09497K.

*RSC Advances* is publishing this expression of concern in order to alert readers that concerns have been raised over the integrity of the data published in this article. The authors have been contacted but have not responded to requests to provide raw data. An expression of concern will continue to be associated with the article until a conclusive outcome is reached.

Laura Fisher, Executive Editor, *RSC Advances*

Date: 31st January 2022

## Supplementary Material

